# Association among dispositional mindfulness, self-compassion, and leukocyte telomere length in Chinese adults

**DOI:** 10.1186/s40359-019-0323-y

**Published:** 2019-07-22

**Authors:** Shian-Ling Keng, Onn Siong Yim, Poh San Lai, Soo Hong Chew, Richard P. Ebstein

**Affiliations:** 1 Division of Social Science, Yale-NUS College, 16 College Ave West, #01-220, Singapore, 138527 Singapore; 2 Department of Paediatrics, National University of Singapore, 21 Lower Kent Ridge Rd, Singapore, 119077 Singapore; 3 Department of Economics, National University of Singapore, 21 Lower Kent Ridge Rd, Singapore, 119077 Singapore; 4 China Center for Behavior Economics and Finance, South Western University of Finance and Economics, Chengdu, China

**Keywords:** Leukocyte telomere length, Cellular aging, Mindfulness, Self-compassion

## Abstract

**Background:**

Whereas meditation training has been purported to support slower cellular aging, little work has explored the association among different facets of dispositional mindfulness, self-compassion, and cellular aging. The present study aimed to examine the relationship between leukocyte telomere length (LTL), an index of cellular aging, dispositional mindfulness, and self-compassion in a sample of Singaporean Chinese adults.

**Methods:**

One hundred and fifty-eight Chinese adults (mean age = 27.24 years; 63.3% female) were recruited from the community and completed self-report measures assessing dispositional mindfulness, self-compassion, and psychological symptoms, as well as provided blood samples for analyses of LTL. Multiple regression analyses were conducted to examine the role of trait mindfulness and self-compassion in predicting LTL, taking into consideration potential covariates such as chronological age and psychological symptoms.

**Results:**

Results showed that nonreactivity, one of the five facets of dispositional mindfulness, was significantly associated with LTL, after controlling for chronological age. There was also a trend for dispositional mindfulness, self-compassion, and their selected facets (i.e., nonjudging, common humanity, and de-identification) to each be associated with longer LTL.

**Conclusions:**

Overall, the findings provide preliminary support for the association among aspects of dispositional mindfulness, self-compassion, and aging. In particular, individuals high on nonreactivity experience slower aging at the cellular level, likely through engaging in more adaptive coping mechanisms.

## Background

In a rapidly aging population, the physiological and psychological mechanisms underpinning successful aging are the focus of intense research. A key biological marker associated with aging at the cellular level is leukocyte telomere length (LTL) [1]. Telomeres cap the ends of chromosomes and serve to protect the chromosomes from deterioration and damage. With increasing cell divisions, telomeres shorten, until a critical length is reached, and the cell enters senescence. LTL is maintained by telomerase, which can add telomeric repeat sequences to the ends of chromosomes, hence elongating them to compensate for their attrition. Since the levels of telomerase are limited in most human tissues [2], over a lifetime of growing and physiological maintenance, the percentage of senescent cells builds up in all tissues, potentially impairing the body’s ability to repair itself. Ipso facto, LTL is widely-employed as a marker for cellular aging and disease [1, 3].

A growing body of research has demonstrated that in addition to chronological age, numerous factors exert effects on telomere length. Shorter telomeres are associated with various chronic illnesses such as cardiovascular diseases [4] and diabetes [5] as well as mental disorders such as major depression [6] and anxiety disorders [7]. More broadly, studies show that shorter LTL is associated with life stressors including urban living and poverty [8]. Shorter LTL has also been linked to impatience, as measured using behavioural economic tasks [9]. Conversely, healthy lifestyles such as exercise are correlated with longer telomeres [10]. Normal personality traits (e.g., the Big Five) have also been examined for correlation with LTL. For example, both neuroticism and hostility have been associated with shorter LTL [11, 12]. Beyond disease factors, lifestyle behaviors, and general personality traits, less work has examined the association between telomere length and putatively *adaptive* psychological traits, such as mindfulness and self-compassion.

Mindfulness, viz. paying attention to experiences in the present moment in an intentional and non-judgmental manner [13], can be conceptualized as a dispositional trait, and is associated with a variety of physical and psychological health outcomes including reduced depression, anxiety, and rumination, as well as improved quality of life and subjective health [14]. As highlighted in a recent review, there is increasing evidence that dispositional mindfulness is an individual trait that is conceptually distinct from other well-established personality traits, such as conscientiousness and neuroticism [15]. Moreover, dispositional mindfulness needs to be considered apart from state mindfulness, which refers to momentary, short term, or current expression of the quality of mindfulness. It is believed that dispositional mindfulness is a natural human capacity, and can be cultivated and strengthened over time through systematic practices such as meditation [16]. A widely-employed measure to operationalize dispositional mindfulness is the five facet mindfulness questionnaire (FFMQ), which describes mindfulness as consisting of five facets (observing, describing, acting with awareness, nonreactivity, nonjudging) [17]. With the exception of the observing facet [18], each of the other facets is consistently associated with psychological well-being [17, 19]. Specifically, it has been demonstrated that the observing facet as assessed in the FFMQ does not include items pertaining to awareness of emotions, which may be crucial to psychological well-being [18]. Taken together, existing research points to the value of examining the role of individual facets of mindfulness as predictors of psychological and physical health.

The positive relationship between mindfulness and psychological well-being suggests the notion that dispositional mindfulness can be pegged to biological markers of aging that correspond with favourable health trajectories [3]. Mindfulness meditation has been theorized to impact biological aging through lowering cognitive stress and stress-related arousal, as well as increasing positive states of mind [20]. Increased positive emotions and lowered stress arousal may result in greater vagal tone and growth hormone axis activity, and lower cortisol, insulin, and oxidative stress, which in turn promote telomere maintenance [3, 20]. In this vein, some investigations, and confirmed by meta-analysis, show that mindfulness training increases telomerase activity [21, 22]. However, there has not been much work examining the association between mindfulness and LTL. One study that examined only a single facet of dispositional mindfulness, mind wandering, found association with shorter LTL in a sample of predominantly Caucasian women [23]. As mindfulness is commonly viewed as a multidimensional construct [17], the involvement of other components of dispositional mindfulness (e.g., the nonreactivity and nonjudging components) crucially need to be evaluated for association with LTL towards a more panoptic understanding of the dynamics of telomere maintenance, dispositional mindfulness, and implications for psychological well-being. Therefore, a key aim of this study was to examine the association between different facets of dispositional mindfulness and LTL in a sample of Chinese adults. Based on prior research, we hypothesized that several attentional (i.e., describe and acting with awareness) and attitudinal (i.e., nonjudging and nonreactivity) facets of trait mindfulness would be associated with longer LTL.

Self-compassion is a personality trait that resonates with mindfulness, and has been associated with adaptive functioning. It refers to the tendency to relate to one’s experiences of pain and suffering with an attitude of kindness and compassion (Neff, 2016), and has been conceptualized as consisting of three aspects: 1) self-kindness, which refers to the ability to relate to oneself kindly; 2) common humanity, which refers to the tendency of perceiving suffering as a common human experience, as opposed to feeling isolated during times of failure; and 3) mindfulness, which refers to being aware of and accepting of one’s inner experiences, versus over-identifying with the experiences. Self-compassion is correlated with greater well-being in both adolescents and older adults [24] and conversely, a meta-analysis found a strong association between low self-compassion and psychopathological symptoms, particularly depression, anxiety, and stress [25]. In a study involving 20 Caucasian Zen meditators, the common humanity subscale of the Self Compassion Scale was found to correlate with improved telomere maintenance [26]. In a second small-scale investigation, also involving a predominantly Caucasian sample, practitioners of loving-kindness meditation, a form of practice that aims to cultivate self-compassion (in addition to compassion towards all living beings), have longer LTL compared to controls [27]. Despite the promising findings suggesting a potential positive association between LTL and compassion-related practices or traits, these studies are limited by small samples and a restricted range of demographics focusing on largely Caucasian participants who are experienced meditators. Further research with bigger and more diverse ethnic and gender cohorts is warranted to gain a fuller understanding of the relationship between facets of self-compassion and LTL.

Surprisingly, the important role of dispositional mindfulness and self-compassion in LTL has yet to be adequately evaluated in the great majority of people who are non-meditators, or do not meditate regularly. Hence, a main goal of the current study is to bridge this critical gap in understanding the dynamics of telomere maintenance, especially the relationship of LTL to psychological health and the personality traits of dispositional mindfulness and self-compassion in a sample of community adults with little or no regular meditation experience. Accordingly, we utilized a cross-sectional design to examine the association between dispositional mindfulness, self-compassion, and LTL in a cohort of Chinese adults of mixed gender. In our analyses, we assessed and controlled for the effects of confounding factors such as chronological age and psychological symptoms (i.e., depression, anxiety, and stress symptoms) on the association between LTL and these personality traits. We hypothesized that both dispositional mindfulness and self-compassion, along with their individual facets, would be associated with longer LTL.

## Methods

### Participants

One hundred and fifty-eight adult participants were recruited via electronic fliers from National University of Singapore and the larger community. Participants were deemed eligible if they were ethnically Chinese, aged between 18 and 55 years old, and had not engaged in mindfulness/meditation practice for more than an average of 20 min a week in the past 6 months. The latter criterion was adopted following Robins, Keng, Ekblad, & Brantley [28] to recruit individuals with no regular meditation or mindfulness practice. Assuming a small-to-medium effect size [23], and using a multiple regression approach, an acceptable power of 0.80 with an alpha of .05 would be achieved with a sample size of approximately 159. Participants were reimbursed SG$35 for their participation. This study was approved by National University of Singapore’s Institutional Review Board.

### Procedure

Data for this study were collected as part of a larger study on the effects of mindfulness training on psychological health. Upon providing informed written consent, participants completed a battery of self-report measures (see below) administered online and provided blood samples. DNA was extracted from whole blood using Qiagen’s QIAamp DNA Blood Midi Kit (Qiagen, USA) spin column centrifugation method. Procedures used were as recommended in manufacturer’s protocols. Purified DNA samples were quantified using a NanoDrop 2000 spectrophotometer (Thermo Fisher Scientific, USA) and stored at − 20 °C before biological assays.

### Measures

#### Demographics

The demographics data form include questions about participants’ age, sex, ethnicity, education background, religion, and marital status.

#### Dispositional mindfulness

The Five Facet Mindfulness Questionnaire (FFMQ) provides a measure of overall dispositional mindfulness and its five facets: describing (the ability to describe one’s internal experiences with words), observing (the ability to observe one’s internal experiences), acting with awareness (the ability to engage in everyday activities in a present moment-centered manner), nonjudging (the ability to be nonjudgmental towards one’s experiences, and nonreactivity (the ability to relate to one’s experiences nonreactively) [17]. The FFMQ comprises 39 items, each rated on a 5-point Likert scale (1 = never or very rarely true; 5 = very often or always true). The scale has demonstrated high internal consistency and construct validity across meditating and non-meditating samples [19]. In the current study, the FFMQ’s overall internal consistency is .87. The internal consistencies of the subscales range between .70 and .90.

#### Self-compassion

The Self-Compassion Scale (SCS) assesses an individual’s tendency to relate to him- or herself in a kind and compassionate manner [29]. The measure consists of 26 items, which correspond to a total of six subscales: 1) self-kindness, 2) self-judgment, 3) common humanity, 4) isolation, 5) mindfulness, and 6) over-identification. The measure was found to demonstrate high internal reliability, test-retest reliability, and factorial validity in a large undergraduate sample [29]. Further, it demonstrated discriminant validity in relation to self-esteem measures. In this sample, the Cronbach’s alpha of the SCS is .82, whereas the internal consistencies of the subscales range from .71 to .81.

#### Depression, anxiety, and stress symptoms

The Depression, Anxiety and Stress Scales-21 (DASS-21) measures the extent to which respondents’ experience symptoms of depression, anxiety, and stress in the past week [30]. Items are rated on a 4-point scale, ranging from 0 (*did not apply to me at all*) to 3 (*applied to me very much or most of the time*). The DASS-21 has demonstrated good convergent and discriminant validity, and also high internal consistencies for all the subscales [31]. In this study, the internal consistencies of the depression, anxiety, and stress subscales are .88, .79, and .83 respectively.

#### Relative leukocyte telomere length (LTL) measurement

Relative LTL was measured using the Cawthon method [32] as previously described by Yim et al. [9]. The method entails amplifying a fixed-length telomere product in a monochrome multiplex polymerase chain reaction (PCR) reaction alongside a single copy reference gene. Normalizing the telomere PCR product to the reference product generates a telomere/single copy (T/S) ratio which is proportional to the average LTL. Quantitative PCR assay for relative LTL shows correlation with the ‘gold-standard’ terminal restriction fragmentation southern blot analyses and is widely adopted for high throughput studies requiring telomere length data. Forty randomly selected samples were used to create a pooled DNA standard of 80 ng/μl, which were then serially diluted to produce standards of 40, 20, 10, 5, 2.5, and 1.25 ng/μl concentrations. Both standards and samples were assayed in triplicates on opaque 96-well plates (Bio-Rad, USA).

The primer sequences used were 5′- ACACTAAGTT TGGGTTTGGG TTTGGGTTTG GGTTAGTGT -3′ & 5′- TGTTAGGTAT CCCTATCCCT ATCCCTATCC CTATCCCTAA CA − 3′ for telomere sequences and 5′- CGGCGGCGGG CGGCGCGGGC TGGGCGGCTT CATCCACGTT CACCTTG -3′ & 5′- GCCCGGCCCG CCGCGCCCGT CCCGCCGGAG GAGAAGTCTG CCGTT − 3′ for haemoglobin beta (HBB) gene as reference. Each reaction contained 8 μl of 2x QuantiFast SYBR Green PCR Master Mix (Qiagen, USA), 1.4 μl of each 10 μM telomere primers, 0.8 μl of each 10 μM reference gene primers, 20 ng of genomic DNA, 0.08 μl of 1 M Dithiothreitol, and sterile water to a total volume of 16 μl. Real-time quantitative polymerase chain reaction protocol: 15 min at 95 °C, 2 cycles of 15 s at 94 °C followed by 15 s at 49 °C, 32 cycles of 15 s at 94 °C, 10 s at 62 °C, 15 s at 74C° with fluorescent signal acquisition, 10 s at 84 °C, and finally 15 s at 88 °C with signal acquisition. PCR was performed on a CFX96 Real-Time PCR Detection System (Bio-Rad, USA).

### Data analytic plan

Analyses were conducted using IBM SPSS. There were no missing data. Data were first checked for normality and outliers. All the variables followed a normal distribution and no transformation was applied. First, we examined the association between LTL, demographic variables, and psychological symptoms. Variables that were significantly correlated with LTL were included as covariates in the subsequent analysis examining the association between LTL, trait mindfulness, and self-compassion. LTL was regressed on total mindfulness score, followed separately by each of the individual facets of mindfulness. The analyses were then repeated by including previously identified covariates in the regression models. The above analyses were repeated for self-compassion and all of its components respectively. To reduce Type I error yet maintain power to detect the association among the variables of interest, we set alpha conservatively at .01.

## Results

### Sample characteristics

The majority of the recruited participants were female (*n* = 100; 63.3%) and single (80.4%). Participants’ mean age was 27.24 years (range = 19–41; SD = 5.24). Most of the participants had a bachelor’s degree (70.3%) or a graduate (master’s or PhD) degree (24.1%), followed by a diploma degree (5.7%). Approximately 21 % of the participants identified as Buddhists, followed by 20.2% who identified as Christians. Thirty-nine percent of the sample denied affiliation with any religion, with the remaining identifying with other religions. Further, 12.7% of the sample reported having received psychological therapy previously, with 1.3% currently attending therapy. Table 1 summarizes the sample characteristics, both overall and stratified by gender. There were no significant gender differences on any of the demographic variables, *p*s > .05. Table 2 lists the descriptive statistics of key study variables.

**Table 1:** Sample Characteristics, Overall and Stratified by Gender

	All Participants (*n* = 158)	Female (*n* = 100)	Male (*n* = 58)
Age M(SD)	27.24 (5.24)	27.28 (5.56)	27.17(4.67)
Marital Status			
Single	127 (80.4%)	84 (84%)	43 (74.1%)
In a Relationship	16 (10.1%)	9 (9%)	7 (12.1%)
Married	14 (8.9%)	7 (7%)	7 (12.1%)
Divorced	1 (.6%)	0	1 (1.7%)
Education			
Diploma	9 (5.7%)	5 (5%)	4 (6.9%)
Bachelor’ Degree	111 (70.3%)	73 (73%)	38 (65.5%)
Post-Graduate Degree (Master’s/PhD)	38 (24.1%)	22 (22%)	16 (27.6%)
Religion*			
No Religion	62 (39.2%)	40 (40%)	22 (37.9%)
Christianity	32 (20.2%)	18 (18%)	14 (24.13%)
Buddhism	34 (21.5%)	20 (20%)	14 (24.13%)
Others	8 (5.1%)	3 (3%)	5 (8.6%)
Previous Experience with Therapy (Yes)	20 (12.7%)	11 (11%)	9 (15.5%)

Note: *the numbers do not add up to 100% due to the presence of missing data across both genders

**Table 2:** Descriptive Statistics of Key Study Variables

Variable	Mean	SD
Leukocyte Telomere Length	1.09	.26
FFMQ-Total	118.96	14.35
FFMQ-Observe	25.11	4.90
FFMQ-Describe	24.71	5.54
FFMQ-Awareness	24.34	5.33
FFMQ-Nonjudging	22.62	5.57
FFMQ-Nonreactivity	21.16	3.35
SCS-Total	3.01	.45
SCS-Self-Kindness	3.10	.79
SCS-Self-Judgment	2.67	.76
SCS-Common Humanity	3.23	.82
SCS-Isolation	3.21	.91
SCS-Mindfulness	3.36	.71
SCS-Overidentification	2.62	.75
DASS-Depression	25.89	8.71
DASS-Anxiety	24.58	7.37
DASS-Stress	29.08	8.20

Note: *FFMQ* = Five Facet Mindfulness Questionnaire, *SCS* = Self-Compassion Scale, *DASS* = Depression, Anxiety, and Stress Scales

### Covariate analyses

**Fig. 1:**
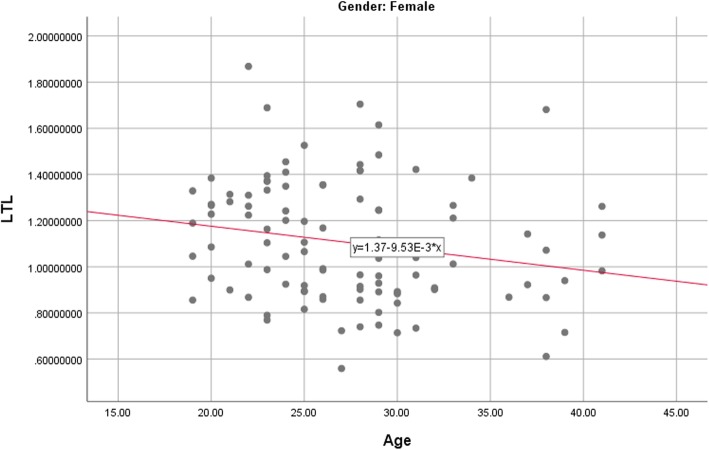
Association between age and LTL (leukocyte telomere length) among female participants

**Fig. 2:**
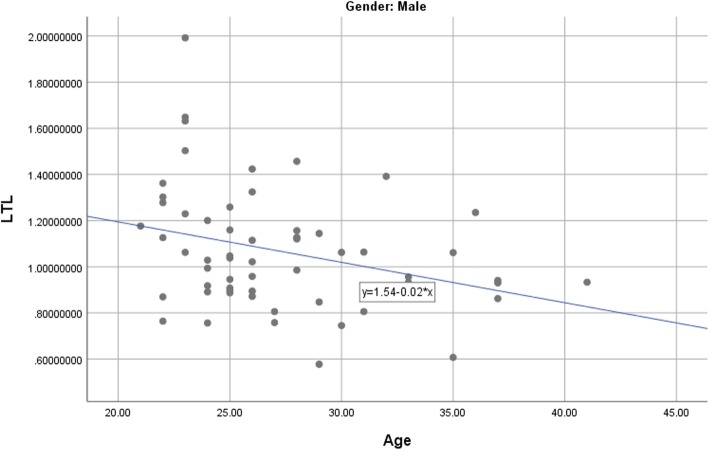
Association between age and LTL (leukocyte telomere length) among male participants

Table 3 presents the correlations between LTL, key demographic variables, and psychological symptoms. Of all the potential covariates, only age was significantly and negatively associated with LTL, *r* = −.23, *p* = .003. The analysis assessing the association between age and LTL was then repeated for each gender respectively. Among male participants, age was significantly associated with LTL, *r* = −.31, *p* = .017. A similar negative association between LTL and age was also found in the female subsample, *r* = −.20, *p* = .046 (see Figs. 1 and 2). Age was therefore included as a covariate in the subsequent primary analyses.

**Table 3:** Correlation between Leukocyte Telomere Length and Potential Covariates

Variable	Pearson’s Correlation
Age	−.23**
Sex	−.07
Education	−.08
Past Therapy Experience	−.09
DASS-Depression	−.10
DASS-Anxiety	−.07
DASS-Stress	−.14

*Note*: *DASS* = Depression, Anxiety, and Stress Scales. **p < .01

### Association between dispositional mindfulness and LTL

Regression analysis showed that total FFMQ score was not associated with LTL, *β* = .12, ΔR^2^ = .02, *p* = .122. We next examined the association between each individual facet of mindfulness and LTL. Of all facets of trait mindfulness, we observed a significant association between nonreactivity and LTL, *β* = .22, ΔR^2^ = .05, *p* = .005. There was no significant association between LTL and any of the other facets of mindfulness, all *p* > .63. Importantly, after including age as a covariate in the regression model, nonreactivity remained significantly and positively associated with LTL, *β* = .21, ΔR^2^ = .05, *p* = .006 (see Table 4). Meanwhile, the association between LTL and total FFMQ score became marginally significant after age was controlled for in the model, *β* = .15, ΔR^2^ = .02, *p* = .057 (see Table 5). There was also a trend for nonjudging to be positively associated with LTL, *β* = .13, ΔR^2^ = .02, *p* = .088 (see Table 6).

**Table 4:** Linear Regression Models with Age and Nonreactivity Predicting Leukocyte Telomere Length

	*B*	*SE*	*β*	*p*
Step 1				
Age	−.012 (−.020, −.004)	.004	−.234	.003
Step 2				
Age	−.011 (−.019, −.004)	.004	−.225	.004
Nonreactivity	.017 (.005, .029)	.006	.213	.006

*Note*: *R*^*2*^ = .055 for Step 1; *ΔR*^*2*^ = .045 for Step 2. 95% biased corrected and accelerated confidence internals are reported in parentheses. Confidence intervals and standard errors are based on 1000 bootstrap samples

**Table 5:** Linear Regression Models with Age and Total FFMQ Score Predicting Leukocyte Telomere Length

	*B*	*SE*	*β*	*p*
Step 1				
Age	−.012 (−.020, −.004)	.004	−.234	.003
Step 2				
Age	−.013 (−.020, −.005)	.004	−.249	.002
Total FFMQ Score	.003 (.000, .006)	.001	.149	.057

*Note*: *R*^*2*^ = .055 for Step 1; *ΔR*^*2*^ = .022 for Step 2. 95% biased corrected and accelerated confidence internals are reported in parentheses. Confidence intervals and standard errors are based on 1000 bootstrap samples. *FFMQ* Five Facet Mindfulness Questionnaire

**Table 6:** Linear Regression Models with Age and Nonjudging Predicting Leukocyte Telomere Length

	*B*	*SE*	*β*	*p*
Step 1				
Age	−.012 (−.020, −.004)	.004	−.234	.003
Step 2				
Age	−.013 (−.021, −.005)	.004	−.254	.001
Nonjudging	.006 (−.001, .014)	.004	.134	.088

*Note*: *R*^*2*^ = .055 for Step 1; *ΔR*^*2*^ = .018 for Step 2. 95% biased corrected and accelerated confidence internals are reported in parentheses. Confidence intervals and standard errors are based on 1000 bootstrap samples

### Association between self-compassion and LTL

Regression analysis showed that there was a trend for total SCS score to be associated with longer LTL, *β* = .18, ΔR^2^ = .03, *p* = .025. Further, two of the subscales of the SCS were positively associated with LTL, albeit a trending association: higher scores on common humanity was associated with longer LTL, *β* = .17, ΔR^2^ = .03, *p* = .038. There was also a positive association between LTL and de-identification from one’s thoughts and emotions (reverse-scored subscale of overidentification), *β* = .07, ΔR^2^ = .01, *p* = .037. None of the other subscales of the SCS were significantly associated with LTL, all *p*s > .16. After controlling for age as a covariate in the regression model, LTL remained positively associated with total SCS score, *β* = .16, ΔR^2^ = .03, *p* = .036 (see Table 7), common humanity, *β* = .17, ΔR^2^ = .03, *p* = .032 (see Table 8), and de-identification from one’s thoughts and emotions, *β* = .18, ΔR^2^ = .03, *p* = .022 (see Table 9). None of the other subscales of the SCS was associated with LTL, *p*s > .19.

**Table 7:** Linear Regression Models with Age and Total SCS Score Predicting Leukocyte Telomere Length

	*B*	*SE*	*β*	*p*
Step 1				
Age	−.012 (−.020, −.004)	.004	−.234	.003
Step 2				
Age	−.011 (−.019, −.004)	.004	−.223	.004
Total SCS Score	.095 (.006, .185)	.045	.163	.036

*Note*: *R*^*2*^ = .055 for Step 1; *ΔR*^*2*^ = .026 for Step 2. 95% biased corrected and accelerated confidence internals are reported in parentheses. Confidence intervals and standard errors are based on 1000 bootstrap samples. SCS = Self-Compassion Scale

**Table 8:** Linear Regression Models with Age and Common Humanity Predicting Leukocyte Telomere Length

	*B*	*SE*	*β*	*p*
Step 1				
Age	−.012 (−.020, −.004)	.004	−.234	.003
Step 2				
Age	−.012 (−.019, −.004)	.004	−.235	.003
Common Humanity	.053 (.005, .102)	.025	.166	.032

*Note*: *R*^*2*^ = .055 for Step 1; *ΔR*^*2*^ = .028 for Step 2. 95% biased corrected and accelerated confidence internals are reported in parentheses. Confidence intervals and standard errors are based on 1000 bootstrap samples

**Table 9:** Linear Regression Models with Age and De-Identification Predicting Leukocyte Telomere Length

	*B*	*SE*	*β*	*p*
Step 1				
Age	−.012 (−.020, −.004)	.004	−.234	.003
Step 2				
Age	−.012 (−.020, −.005)	.004	−.243	.002
De-Identification	.063 (.009, .116)	.027	.178	.022

*Note*: *R*^*2*^ = .055 for Step 1; *ΔR*^*2*^ = .032 for Step 2. 95% biased corrected and accelerated confidence internals are reported in parentheses. Confidence intervals and standard errors are based on 1000 bootstrap samples

## Discussion

Our study finds that nonreactivity, one of the core facets of mindfulness, is significantly correlated with longer LTL in a sample of Chinese adults, with the association remaining even after controlling for chronological age. Further, there was a trend for dispositional mindfulness and nonjudging to be associated with longer LTL. There was also a marginally significant positive association between self-compassion and its selected components (i.e., common humanity and de-identification) and LTL. To our knowledge, this study is among the first to explore and demonstrate the association among dispositional mindfulness, self-compassion, and LTL in an Asian sample and context.

The finding that nonreactivity is associated with longer LTL in our sample highlights the salient role of attitudinal dimensions of mindfulness in promoting slower aging at the cellular level. This corresponds with theoretical conceptualizations of mindfulness as a state that facilitates decentering and more adaptive appraisal of stress-inducing situations [20, 33]. It is likely that people who are less reactive to their internal experiences are more able to tolerate distress without engaging in maladaptive cognitive styles (e.g., rumination) or behaviours (e.g., avoidance), which exacerbate the psychological and physiological effects of stress. The association between nonreactivity and LTL remains significant after controlling for age. The finding extends the results of a study by Alda and colleagues [26], who found a positive association between acceptance of one’s inner experiences and LTL in a small sample of meditators compared to a control group. The finding suggests that physical and behavioural health may be influenced more by how people, including those who have not engaged in regular meditation, relate to their internal experiences. Notably, the ‘how’ of relating to internal experiences can mitigate the wear and tear of negative psychological symptoms in nonmeditators on aging at the cellular level, as indexed by LTL. Alternatively, it is plausible that people with longer LTL tend to be less reactive to their internal experiences generally. Further research is need to clarify the nature and direction of the association between LTL and nonreactivity.

It is intriguing that other facets of mindfulness are either not associated, or weakly associated with LTL. For example, there was a trend for both dispositional mindfulness and nonjudging to be associated with longer LTL when age was controlled for. The findings suggest that of all facets of mindfulness, it is the nonreactivity and nonjudging facets, as opposed to other facets that correspond to the attentional dimensions of mindfulness (e.g., observe and describe), which contribute the most to the association between mindfulness and cellular aging. The findings correspond to existing research demonstrating that nonreactivity and nonjudging tend to constitute among the greatest variances in predicting psychological symptoms and well-being across both clinical and nonclinical samples [34, 35].

The present study is among the first to explore the association between self-compassion and LTL in a general community sample without regular meditation practice experience. The findings that self-compassion and its selected facets were each related to longer LTL (albeit nonsignificant following the application of Type 1 error correction) are consistent with the idea that self-compassion facilitates a kinder and more adaptive way of relating to one’s experiences, which helps mitigate age-associated telomere erosion. Whereas Alda et al. [26] studied experienced meditators and found results consistent with our findings, the current study highlights the potential role of self-compassion as a mitigator of cellular aging in a community sample. Meanwhile, it is plausible that individuals with longer LTL tend to have a kinder disposition towards themselves and the world. For example, past research has demonstrated that men with shorter telomere length have higher levels of hostility (which can be considered as the opposite of having a compassionate disposition) compared to those with longer telomeres [11]; however, like many other studies examining psychological correlates of LTL, the cross-sectional nature of the study limits the interpretation of causality. Future research would benefit from exploring potential mechanisms that may underlie the association between self-compassion and LTL. One intriguing possibility would be to examine whether lifestyle or behavioral interventions known to impact telomere dynamics (e.g., physical exercise) may simultaneously result in changes in psychological traits, such as self-compassion and mindfulness [36].

It is notable that the present study did not find a direct association between LTL and measures of psychological symptoms, which is contrary to other studies that have demonstrated a negative association between LTL and psychological distress [37, 38]. However, there was a marginally significant negative association between stress and LTL. One reason underlying the above discrepancy may be due to the fact that other studies tend to measure extreme states of psychological symptoms by including clinical populations with psychiatric disorders (e.g., patients with major depressive disorder) [39] or examining specific populations under stressful contexts (e.g., caregivers of patients with Alzheimer’s Disease) [40]. The fact that our study recruited a nonclinical sample likely results in a dataset with less severe and a more limited range of psychological symptoms, which precludes our ability to detect a significant relationship between LTL and psychological symptoms. For example, the mean scores on the depression and stress subscales of the DASS-21 in our sample are both in the mild range of severity.

The present study is characterized by several strengths, including recruitment of a relatively large and ethnically homogenous Chinese sample, and adoption of a multidimensional measurement of mindfulness and self-compassion. Our analyses carefully assessed and controlled for potential confounding factors known to be associated with telomere length, such as chronological age. Given that existing research on psychological correlates of LTL tend to focus on Western samples, the findings also contribute to a greater understanding of the association between telomere biology and psychological traits in an Asian context. A weakness of the study is the cross-sectional design, which limits the inference of causality. Future research should adopt a randomized controlled design to examine whether mindfulness and self-compassion training could lead to changes in LTL (or, as stated above, whether other interventions designed to enhance telomere maintenance might lead to simultaneous changes in mindfulness and self-compassion). Also, it would be worthwhile to employ mediational analyses to examine psychological and biological pathways, such as maladaptive coping styles (e.g., rumination and avoidance), sympathetic nervous system arousal, and genetic markers that may mediate the role of psychological traits on LTL [9, 20]. The fact that our sample consisted of relatively young adults motivated to engage in mindfulness training also precludes generalizability of the findings to the larger population. Future research should aim to recruit a larger, ideally unselected sample that is more representative of the population. Also, we did not assess and control for past experience with meditation practice in the analysis; future research would benefit from assessing the extent to which amount of past meditation experience may influence the association among trait mindfulness, self-compassion, and LTL. Further, telomerase activity, another known marker of cellular aging, was not assessed in this study. This is due to the fact that we analyzed peripheral blood cells, the most convenient source of tissue for most studies, which contain low levels of telomerase activity [41]. While telomerase activity can be measured, the low levels make it technically challenging to optimize the experiments required for the assessment. Relative to telomerase activity, telomere length is influenced by a broader range of factors, including genetic, cellular, and environmental factors [42]. Therefore, we deem telomere length to be a better biomarker in assessing the association between behavioural traits and aging. Lastly, there is growing evidence that immune cell types vary in telomere length, which may contribute differentially to LTL estimates [20]. As our study did not assess the distribution of specific cell types, we cannot rule out the possibility that systematic differences in cell distribution may account for the observed association between LTL and behavioural traits. Future research should assess ways in which variations in cell distribution may influence estimates of LTL.

## Conclusions

In conclusion, the present study is, to our knowledge, the first to demonstrate associations between aspects of dispositional mindfulness, self-compassion, and LTL in a community sample of Chinese adults who do not meditate regularly. The findings highlight the role of nonreactivity as a protective factor of the telomere maintenance system, and future research is required to clarify the causal mechanisms underlying the association among LTL, dispositional mindfulness, and self-compassion.

## Data Availability

The datasets used and/or analyzed during the current study are available from the corresponding author on reasonable request.

## Abbreviations

DASS-21: Depression, Anxiety and Stress Scales-21
FFMQ: Five Facet Mindfulness Questionnaire
LTL: Leukocyte Telomere Length
PCR: Polymerase Chain Reaction
SCS: Self-Compassion Scale
SPSS: Statistical Package for the Social Sciences
