# Correction to: Association among dispositional mindfulness, self-compassion, and leukocyte telomere length in Chinese adults

**DOI:** 10.1186/s40359-020-00433-1

**Published:** 2020-06-22

**Authors:** Shian-Ling Keng, Onn Siong Yim, Poh San Lai, Soo Hong Chew, Richard P. Ebstein

**Affiliations:** 1 Division of Social Science, Yale-NUS College, 16 College Ave West, #01-220, Singapore, 138527 Singapore; 2 Department of Paediatrics, National University of Singapore, 21 Lower Kent Ridge Rd, Singapore, 119077 Singapore; 3 Department of Economics, National University of Singapore, 21 Lower Kent Ridge Rd, Singapore, 119077 Singapore; 4 China Center for Behavior Economics and Finance, South Western University of Finance and Economics, Chengdu, China

**Correction to: BMC Psychol 7, 47 (2019)**

**https://doi.org/10.1186/s40359-019-0323-y**

Following publication of the original article [1], the authors reported an error in coding of the Isolation subscale of the Self-Compassion Scale.

Namely, the mean score for SCS-Isolation reported in Table 2 should be 2.79 (SD = .91). Following this correction, the mean score of the overall SCS scale should be 2.96 (SD = .59).

Re-analyses using the newly computed scores showed there was a trend for self-compassion to be positively associated with leukocyte telomere length (LTL), β = .13, Δ*R*^2^ = .02, *p* = .10. The trend remains after including age as a covariate in the analyses, β = .13, Δ*R*^2^ = .02, *p* = .09. The association between the isolation subscale and LTL remained insignificant (with or without including age as a covariate) following the re-analyses.

The authors thank you for reading this correction and apologize for any inconvenience caused.
